# Effects of pharmacogenetic profiles on pediatric pain relief and adverse events with ibuprofen and oxycodone

**DOI:** 10.1097/PR9.0000000000001113

**Published:** 2023-10-17

**Authors:** Samina Ali, Aran Yukseloglu, Colin J. Ross, Rhonda J. Rosychuk, Amy L. Drendel, Robin Manaloor, David W. Johnson, Sylvie Le May, Bruce Carleton

**Affiliations:** a Department of Pediatrics, Faculty of Medicine & Dentistry and Women & Children's Health Research Institute (WCHRI), University of Alberta, Edmonton, AB, Canada; b Faculty of Pharmaceutical Sciences, University of British Columbia, Vancouver, BC, Canada; c Department of Pediatrics, Medical College of Wisconsin, Milwaukee, WI, USA; d Department of Anaesthesiology, College of Medicine, University of Saskatchewan, Saskatoon, SK, Canada; e Department of Pediatrics, Alberta Children's Hospital, University of Calgary, Calgary, AB, Canada; f Faculty of Nursing, Université de Montréal, CHU Sainte-Justine Research Centre, Montreal, QC, Canada; g Division of Translational Therapeutics, Department of Pediatrics, Faculty of Medicine, University of British Columbia, Vancouver, BC, Canada

**Keywords:** Pharmacogenomics, Opioids, Analgesia, Children, Side effects

## Abstract

**Introduction:**

Individual genetic variation may influence clinical effects for pain medications. Effects of CYP2C9, CYP3A4, and CYP2D6 polymorphisms on clinical effectiveness and safety for ibuprofen and oxycodone were studied.

**Objective:**

Primary objectives were to AU2 evaluate if allelic variations would affect clinical effectiveness and adverse events (AEs) occurrence.

**Methods:**

This pragmatic prospective, observational cohort included children aged 4 to 16 years who were seen in a pediatric emergency department with an acute fracture and prescribed ibuprofen or oxycodone for at-home pain management. Saliva samples were obtained for genotyping of allelic variants, and daily telephone follow-up was conducted for 3 days. Pain was measured using the Faces Pain Scale-Revised.

**Results:**

We included 210 children (n = 140 ibuprofen and n = 70 oxycodone); mean age was 11.1 (±SD 3.5) years, 33.8% were female. Median pain reduction on day 1 was similar between groups [ibuprofen 4 (IQR 2,4) and oxycodone 4 (IQR 2,6), *P* = 0.69]. Over the 3 days, the oxycodone group experienced more AE than the ibuprofen group (78.3% vs 53.2%, *P* < 0.001). Those with a CYP2C9*2 reduced function allele experienced less adverse events with ibuprofen compared with those with a normal functioning allele CYP2C9*1 (*P* = 0.003). Neither CYP3A4 variants nor CYP2D6 phenotype classification affected clinical effect or AE.

**Conclusion:**

Although pain relief was similar, children receiving oxycodone experienced more AE, overall, than those receiving ibuprofen. For children receiving ibuprofen or oxycodone, pain relief was not affected by genetic variations in CYP2C9 or CYP3A4/CYP2D6, respectively. For children receiving ibuprofen, the presence of CYP2C9*2 was associated with less adverse events.

## Abstract 2

Supplemental Digital Content is Available in the Text.

In a pediatric cohort with fracture, genotype did not alter analgesic effect for ibuprofen or oxycodone, however CYP2C9 variation influenced adverse effects for ibuprofen.

## 1. Introduction

Pain experiences can be highly variable between individuals and a standardized approach may not be sufficient, or even safe, for all patients.^12,17^ Genetic differences affected not only pain perception and efficacy, but also the likelihood to experience adverse events related to analgesic use.^26^ Pharmacogenetics can help explain these differences and how to tailor treatment accordingly. Cytochrome enzymes can be highly variable, with numerous single nucleotide polymorphisms (SNPs) known to impact response to medication.^14,15^ Single nucleotide polymorphism variation can result in a variety of metabolic phenotypes, with corresponding pharmacokinetic differences affecting clinical drug effectiveness and safety for individual patients.

Nonsteroidal anti-inflammatory drugs (NSAIDs) and opioids are 2 of the most commonly used categories of pain medications in the world.^30^ Nonsteroidal anti-inflammatory drugs such as ibuprofen are primarily metabolized by CYP2C9 to inactive metabolites^2,6,25^; SNPs affecting these genes can reduce ibuprofen clearance.^31^ Reduced ibuprofen clearance may result in more active serum drug levels which can increase clinical effectiveness but also may increase the risk of adverse events. Oxycodone, a frequently prescribed oral opioid, is known to be primarily metabolized by CYP3A4 and CYP2D6 to metabolites of varying activity.^23^ CYP3A4 is involved in metabolizing around 50% of currently prescribed medications and is the primary metabolizer of oxycodone.^12,47^ CYP2D6 metabolizes approximately 25% of all clinically used medications^6^ and is responsible for conversion of oxycodone to its most active metabolite, oxymorphone.^5,29^ Allelic variants of CYP2D6 lead to different opioid-related metabolic phenotypes, ranging from poor metabolism to ultrarapid metabolism,^6^ which affects analgesic effect and risk for opioid toxicity for individual patients.^28,29^ Unmetabolized oxycodone is responsible for most of its analgesic activity.

This study's goal was to determine how metabolic function of CYP2C9 or CYP3A4/CYP2D6 would affect the clinical effectiveness or safety profile of ibuprofen or oxycodone, respectively. We hypothesized that reduced function of CYP2C9 would lead to increased effectiveness and more adverse events with ibuprofen, reduced functioning of CYP3A4 would lead to increased analgesic effectiveness and increased adverse events, and reduced function of CYP2D6 would lead to reduced effectiveness and decreased adverse events with oxycodone.

## 2. Methods

### 2.1. Study design and setting

This study was a pragmatic prospective, observational cohort study conducted in the Emergency Department (ED) of the Stollery Children's Hospital (Edmonton, Alberta, Canada), a tertiary care facility with 163 beds. Ethics approval was obtained through the University of Alberta's Health Research Ethics Board (Pro00005942).

### 2.2. Participants

Children were eligible if aged 4 to 16 years and being discharged from the ED with an acute, nonoperative fracture and a recommendation to use either ibuprofen or oxycodone for pain management. To be included in the analyses relating genomic associations with clinical outcomes, participants required a DNA sample to be obtained and they must have taken their prescribed medication on day 1 following ED discharge. Exclusion criteria included baseline daily use of medications for chronic pain, being prescribed both ibuprofen and oxycodone on discharge, cognitive impairment preventing self-reporting of pain, inability of family to communicate in English, and no telephone access to the family. The use of adjuvant acetaminophen at the family's discretion, while not explicitly encouraged, was not prohibited for the ethical reason of avoiding untreated pain in participants.

### 2.3. Outcomes

The primary genetic outcome was the frequency of known CYP2C9, CYP3A4, and CYP2D6 allelic variants relevant to ibuprofen and oxycodone metabolism. The primary clinical outcome measures were postmedication use pain score reduction and occurrence of any adverse event on days 1 to 3 after ED discharge. Pain scores were measured with the Faces Pain Scale-Revised (FPS-R),^22^ a validated 6-item self-report tool. The following child-reported daily pain score measurements were collected for 3 consecutive days after ED discharge: average pain, minimum pain, maximum pain (that was treated), and postmedication administration pain. For the purposes of this study, pain reduction was defined as the difference between maximal pain and postmedication administration pain on day 1 and was treated as a continuous variable. Adverse event (AE) measurements on days 1 to 3 were categorized as present/absent for occurrence of any adverse event on that day and were coded according to MedDRA (https://www.meddra.org/).^24^ CYP3A4 and CYP2C9 allelic variants were classified as present or absent. Variants of interest included CYP3A4 *8, *11, *13, and *17; CYP2C9 *1, *2, *3; and *6. CYP2D6 allelic variants were scored, and metabolic phenotype classified based on the previously described and recently updated classification: poor (score 0), intermediate (score 0.25–1), normal (score 1.25–2.25), and ultrarapid (score >2.25) metabolic phenotypes.^10^

### 2.4. Recruitment

All caregivers provided written informed consent and children >6 years, written assent. Trained research assistants confirmed eligibility, collected basic demographic and diagnostic data, completed a medical record review, and procured saliva samples while the patient was in the ED. Oragene (Ottawa, Canada) saliva tube collection kits were used; if the child was unable to produce required saliva volumes spontaneously, saliva sponge swab sticks were used. Saliva samples were transported in preservative and sent by courier to the Canadian Pharmacogenomic Network for Drug Safety (Vancouver, British Columbia) for DNA analyses.^9^

Participants received a copy of the FPS-R for reference and a logbook on discharge. In the following 3 days, caregivers received daily phone calls during which they were asked to provide their child's *self-reported* FPS-R pain score for average, maximal, minimal, and postmedication treatment pain. These calls also included questions about home medication use, nonpharmacologic management, adverse events, and functional outcomes. Furthermore, phone calls at 2-week and 6-week postinjury collected details regarding need for medical follow-up.

### 2.5. Data collection and management

All data were entered into OpenClinica by a data entry specialist, who validated data on system entry to minimize errors.^33^ Adverse events data, including side effects, were coded and recorded using MedDRA, a Health Canada recommended terminology for reporting of adverse events (https://www.meddra.org/).^24^

DNA samples were genotyped for specific gene variants in *CYP2C9*, *CYP3A4*, *and CYP2D6* with a custom absorption, distribution, metabolism, and excretion (ADME) panel of variants using an Illumina Infinium assay (Illumina, San Diego, CA) and selected CYP2D6 variants by SNaPshot Assay, as previously described.^40^ The polymorphisms analyzed were CYP2C9 (*1, *2, *3, *5, *6), CYP2D6 (*1, *2, *3, *4, *5, *6, *9, *10, *17, *41), and CYP3A4 (*1B, *2, *3, *4, *7, *8, *11, *13, *14, *16, *17, *18, *19, 11460A>G(K96E), 14313G>A(E174H), 16907T>G(S252A), 20239G>A, 23139T>C (I431T)). Staff-performing DNA analyses were blinded to clinical results.

### 2.6. Sample size and power

This paper is an exploratory secondary analysis of data collected for a larger observational cohort study.^3^ As such, no specific power calculations were conducted for this study.

### 2.7. Statistical methods

Analyses were conducted for each medication group separately. Descriptive statistics included mean, SD, counts, and percentages. T tests and χ^2^ tests assessed mean differences and frequency of AEs between medication groups. Allele-based regression models with only genomic haplotypes as predictors were used to determine if any allele variants within the study population were associated with pain relief or adverse events. For pain reduction, the multiple linear regression model also included variables age, sex, fracture location, fracture reduction, nonpharmacologic management, and ethnicity to provide adjusted estimates. For adverse events, the logistic regression model had variables age, sex, fracture location, sedation in ED, and ethnicity. Linear regression estimates and associated coefficient estimates and standard errors were calculated, as well as odds ratios (ORs) for logistic regression analyses. Statistical analyses were performed in R with missing data removed from tests and regressions; a *P*-value <0.05 was considered statistically significant.^37^

## 3. Results

### 3.1. Demographic characteristics

Of the 329 children enrolled in the original study,^2^ a total of 210 (n = 140 ibuprofen and n = 70 oxycodone) had DNA analyses available and were included in this study. Mean age was 11.1 (SD 3.5) years, with 33.8% of the participants being female, and 79.0% self-identifying as Anglo-American (Table 1).

**Table 1:** Demographic characteristics (n = 210).*

	Ibuprofen group (n = 140)	Oxycodone group (n = 70)	Total cohort (n = 210)
Age, mean (SD), y	10.4 (3.7)	12.5 (2.7)	11.1 (3.5)
Weight, mean (SD), kg	40.9 (17.2)	48.6 (15.1)	43.5 (16.9)
Gender (female), n (%)	56 (40.0)	15 (21.4)	71 (33.8)
Origin of family, n (%)			
Anglo-American	108 (77.9)	58 (82.9)	166 (79.0)
Indigenous	6 (4.3)	3 (4.3)	9 (4.3)
Asian	6 (4.3)	2 (2.9)	8 (3.8)
Hispanic	4 (2.9)	2 (2.9)	6 (2.9)
Métis	2 (1.4)	2 (2.9)	4 (1.9)
Arab	3 (2.1)	1 (1.4)	4 (1.9)
Black	2 (1.4)	1 (1.4)	3 (1.4)
East Indian	2 (1.4)	1 (1.4)	3 (1.4)
Others†	3 (2.1)	0 (0.0)	3 (1.4)
Declined to answer	3 (2.1)	0 (0.0)	3 (1.4)
Fracture location, n (%)			
Upper limb	109 (77.9)	57 (82.6)	166 (79.4)
Lower limb	31 (22.1)	12 (17.4)	43 (20.5)
Procedural sedation, n (%)	42 (30.0)	24 (34.8)	66 (31.6)
Fracture reduction, n (%)	47 (33.6)	25 (36.2)	72 (34.4)
Buckle fracture, n (%)	16 (11.4)	2 (2.9)	18 (8.6)
Discharge plan, n (%)			
Follow-up with the orthopedic surgeon	101 (72.1)	56 (80.0)	157 (74.8)
Follow-up with the family doctor	12 (8.6)	9 (12.9)	21 (10.0)
Referral to the plastic surgeon	4 (2.9)	2 (2.9)	6 (2.9)
Return to ED, as needed	2 (1.4)	1 (1.4)	3 (1.4)
Return to ED, scheduled	3 (2.1)	0 (0.0)	3 (1.4)
Not charted/unable to ask MD	18 (12.9)	2 (2.9)	20 (9.5)

* Number of respondents varied per question.

† Includes “African/Anglo-American,” “Anglo-American/Filipino,” and “Anglo-American/Arab.”

ED, emergency department.

### 3.2. Sample distribution of genetic polymorphisms

Overall, 27.2% (38/140) of children had an allelic variation known to be implicated in clinical outcomes for CYP2C9 and 7.2% (4/57) for CYP3A4, whereas 36.5% (23/63) had “nonnormal” phenotype classification for CYP2D6. CYP2C9 alleles with normal function were found in 72.9% of participants, whereas 26.5% had reduced function, and 0.7% had nonfunctional alleles associated. For CYP3A4, 7.2% of participants' alleles were known to have reduced function; those without variants found were classified as normal functioning wild-type. For CYP2D6, 7.9% of participants were phenotypically classified as poor metabolizers, 28.6% intermediate metabolizers, and 63.5% were normal metabolizers (Table 2).

**Table 2:** Distribution of genetic polymorphisms with known functionality.

CYP2C9 allele function	Allele frequency in the ibuprofen group (n = 140) n (%)
Normal function	
*1	102 (72.9)
Decreased function	
*2	25 (17.9)
*3	12 (8.6)
Nonfunctional	
*6	1 (0.7)
CYP3A4 allele function	Allele frequency in the oxycodone group (n =57) n (%)
Decreased function	
*8	1 (1.8)
*11	1 (1.8)
*13	1 (1.8)
*17	1 (1.8)
CYP2D6 metabolic phenotype (Score)†	Phenotype frequency in the oxycodone group (n = 63) n (%)
Poor (0)	5 (7.9)
Intermediate (0.25–1)	18 (28.6)
Normal (1.25–2.25)	40 (63.5)
Ultrarapid (>2.25)	0 (0)

Based on alleles tested, those without any variants identified were classified to be wild type: *1A. Only alleles with known functionality are presented in this table; thus, proportions are not additive to 100%.

† Based on: Caudle KE, Sangkuhl K, Whirl-Carrillo M, et al. Standardizing CYP2D6 Genotype to Phenotype Translation: Consensus Recommendations from the Clinical Pharmacogenetics Implementation Consortium and Dutch Pharmacogenetics Working Group. Clin Transl Sci. 2020;13(1):116–124. doi:10.1111/CTS.12692.

Of note, 11 participants had copy number variations in CYP2D6; it was not possible for our laboratory to analyze which allele was duplicated, and thus, they were removed from regression analyses and Table 2, and are described narratively, instead. Six of these patients were ultrarapid metabolizers with phenotypic activity scores >3.0. Three of these patients had activity scores between 2 and 2.5; classified as normal or ultrarapid metabolizers depending on the allele that was duplicated. Two of these patients had activity scores between 1 and 2, classified as intermediate or normal metabolizers depending on the allele that was duplicated.

### 3.3. Pain management

Using an “intention to treat” principle, pain scores, including maximum, minimum, average, postmedication treatment pain, and pain reduction on days 1 to 3, were not clinically different between the ibuprofen and oxycodone groups (Table 3).

**Table 3:** Median pain scores, days 1–3.

	Day 1		Day 2		Day 3	
	Ibuprofen group (n = 132)	Oxycodone group (n = 69)	Ibuprofen group (n = 124)	Oxycodone group (n = 64)	Ibuprofen group (n = 102)	Oxycodone group (n = 57)
Maximum pain Median (Q1,Q3)	6 (4,8)	6 (4,8)	6 (4,8)	6 (4,8)	4 (2,6)	6 (2,6)
Posttreatment pain Median (Q1,Q3)	2 (0,4)	2 (1,4)	2 (0,4)	2 (0,4)	2 (0,2)	2 (0,4)
∆ pain* Median (Q1,Q3)	4 (2,4)	4 (2,6)	4 (2,4)	4 (2,4)	4 (2,4)	4 (2,4)
Minimum pain Median (Q1,Q3)	2 (0,2)	2 (2,4)	2 (0,2)	2 (2,2)	0 (0,2)	2 (0,2)
Average daily pain Median (Q1,Q3)	4 (2,6)	4 (2,6)	4 (2,4)	4 (2,6)	2 (2,4)	2 (2,4)

* ∆ Pain is defined as maximum pain − posttreatment pain.

Pharmacologic pain management use on day 1 revealed exclusive ibuprofen use in 99.3% of the ibuprofen group. For the oxycodone group, 85.7% used an oxycodone-based therapy, with 18.6% using oxycodone monotherapy and 67.1% using a commercially available oxycodone/acetaminophen combination tablet on day 1 (Supplemental Table 1, http://links.lww.com/PR9/A212). Mean dose of administered ibuprofen was 9.2 mg/kg/dose (SD 3.5) on day 1, 9.0 mg/kg (SD 3.8) on day 2, and 8.5 mg/kg (SD 3.8) on day 3; of note, these are all within the recommended dosing range of 5 to 10 mg/kg/dose. Mean dose of administered oxycodone was 0.11 mg/kg/dose (SD 0.05) on day 1, 0.10 mg/kg (SD 0.04) on day 2, and 0.09 mg/kg (SD 0.04) on day 3; these are all within the recommended dosing range of 0.05 to 0.15 mg/kg/dose.

Nonpharmacologic pain management is presented in Table 4. On day 1, 67.9% of the ibuprofen group and 57.1% of the oxycodone group used casts, whereas 15% and 2.9%, respectively, used splints; these were considered therapeutic treatment for their fractures. Other nonpharmacologic strategies such as ice, tensor, and crutches were used as adjuvants.

**Table 4:** Nonpharmacological management used (multiple responses permitted).*

	Ibuprofen group (n = 140) n (%)				Oxycodone group (n = 70) n (%)				Total cohort (n = 210) n (%)			
	Used in ED	Day 1	Day 2	Day 3	Used in ED	Day 1	Day 2	Day 3	Used in ED	Day 1	Day 2	Day 3
None	2 (1.4)	4 (2.9)	7 (5.0)	3 (2.1)	0 (0.0)	2 (2.9)	1 (1.4)	3 (4.2)	2 (1.0)	6 (2.9)	8 (3.8)	6 (2.9)
Splint	39 (27.9)	21 (15.0)	21 (15.1)	22 (16.2)	6 (8.6)	2 (2.9)	1 (1.4)	6 (9.4)	45 (21.4)	23 (11.0)	22 (10.5)	28 (14.0)
Cast	89 (63.6)	95 (67.9)	88 (63.3)	88 (64.7)	36 (51.4)	40 (57.1)	39 (55.7)	31 (48.4)	125 (59.5)	135 (64.3)	127 (60.8)	119 (59.5)
Sling	35 (25.0)	42 (30.0)	37 (26.6)	36 (26.5)	36 (51.4)	34 (48.6)	37 (52.9)	29 (45.3)	71 (33.8)	76 (36.2)	74 (35.4)	65 (32.5)
Ice	6 (4.3)	23 (16.4)	23 (16.5)	14 (10.3)	6 (8.6)	14 (20.0)	24 (34.3)	10 (15.6)	12 (5.7)	37 (17.6)	47 (22.5)	24 (12.0)
Tensor	0	2 (1.4)	0	0	2 (2.9)	0	3 (4.3)	2 (3.1)	2 (1.0)	2 (1.0)	3 (1.4)	2 (1.0)
Elevation	3 (2.1)	47 (33.6)	41 (29.5)	39 (28.9)	1 (1.4)	13 (18.6)	9 (12.9)	9 (14.1)	4 (1.9)	60 (28.6)	50 (23.9)	48 (24.0)
Wheelchair	0	0	0	1 (0.7)	0	0	1 (1.4)	1 (1.6)	0	0	1 (0.5)	2 (1.0)
Crutches	6 (4.3)	2 (1.4)	1 (0.7)	0	4 (5.7)	2 (2.9)	2 (2.9)	2 (3.1)	10 (4.8)	4 (1.9)	3 (1.4)	2 (1.0)

* Number of respondents varied per day.

ED, emergency department.

### 3.4. Adverse events

Table 5 shows cumulative presence of adverse events throughout days 1 to 3. Cumulatively over days 1 to 3, 69.8% (143/208) of all children reported at least one adverse event; specifically, 60.4% (84/139) in the ibuprofen group and 89.3% (59/66) in the oxycodone group (*P* < 0.001). On day 1, 61.6% (128/208) of all children reported at least one adverse event; specifically, 52.9% (74/140) in the ibuprofen group and 78.3% (54/69) in the oxycodone group (*P* < 0.001). The ibuprofen group experienced significantly less appetite loss (*P* = 0.04), constipation (*P* = 0.002), dizziness (*P* < 0.001), drowsiness (*P* = 0.001), and nausea (*P* < 0.001) than the oxycodone group. On day 2, 42.7% (88/206) of all children reported adverse events, with 35.7% (50/140) in the ibuprofen group and 57.6% (38/66) in the oxycodone group (*P* = 0.003). On day 3, 27.0% (55/204) of all children reported adverse events, with 22.1% (31/140) in the ibuprofen group and 32.8% (21/64) in the oxycodone group (*P* = 0.11) (Supplemental Table 2, http://links.lww.com/PR9/A212).

**Table 5:** Cumulative adverse events per patient by the treatment group.*

	Ibuprofen Group, n (%) (n = 140)	Oxycodone group, n (%) (n = 69)	Total, n (%) (n = 209)	*P*
Any AE	84 (60.4)	59 (89.3)	143 (69.8)	<0.001
Abdominal pain	11 (7.9)	12 (18.2)	23 (11.2)	0.0224
Appetite loss	44 (31.7)	31 (46.9)	75 (36.6)	0.0152
Constipation	14 (10.1)	15 (22.8)	29 (14.1)	0.0099
Dizziness	13 (9.4)	24 (36.7)	37 (18.0)	<0.001
Drowsiness	64 (46.0)	47 (71.2)	111 (54.1)	<0.001
Nausea	18 (12.9)	27 (40.9)	45 (22.0)	<0.001
Rash	4 (2.9)	4 (6.1)	8 (3.9)	0.2368
Vomiting	3 (2.2)	11 (16.7)	14 (6.8)	<0.001

* Number of respondents varied per day.

AE, adverse events.

### 3.5. Analyses with genomic predictors only

For the ibuprofen group, children who were carriers of the CYP2C9*2 reduced function allelic variant were less likely to experience an adverse event (OR = 0.72, 95% confidence interval [CI] [0.58, 0.89]), compared with children who were carriers of the normal or wildtype CYP2C9*1 allele. In the ibuprofen group, the presence of reduced function CYP2C9*2, CYP2C9*3, and nonfunctional CYP2C9*6 did not influence pain reduction on day 1. For the oxycodone group, no influence was found on pain relief or adverse events, based on CYP3A4 variants and CYP2D6 phenotype classification (Supplemental Tables 3a and 3b, http://links.lww.com/PR9/A212).

### 3.6. Multivariable analyses

For the ibuprofen group, multivariable logistic regression analyses were conducted for pain reduction on day 1, including the independent clinical variables of age, sex, fracture location, fracture reduction, nonpharmacologic management (ie, ice, elevation, and tensor bandage), ethnicity, and CYP2C9*2, *3, and *6 variants; none demonstrated statistical significance.

For the oxycodone group, linear regression analysis for pain reduction on day 1 included the same clinical variables and CYP2D6 intermediate and extensive metabolizer phenotypes. The use of nonpharmacologic pain management strategies (*P* = 0.02) was associated with less pain relief for children using oxycodone. Linear regression analysis for pain reduction on day 1 was also performed for CYP3A4, with the same clinical variables; nonpharmacologic pain management strategies (*P* = 0.006) were associated with less pain relief when using oxycodone.

For the ibuprofen group, multivariable logistic regression analysis for adverse events on day 1 included age, sex, fracture location, procedural sedation in ED, and ethnicity, along with CYP2C9*2, *3, and *6 variants. Children with the CYP2C9*2 allele were less likely to experience adverse events (OR = 0.72, 95% CI [0.58, 0.90]) compared with those with the CYP2C9*1 (wildtype) allele. For the oxycodone group CYP2D6 analysis, the same clinical variables were used, plus intermediate and extensive metabolizer phenotypes were included in the model (Supplemental Tables 4a and 4b, http://links.lww.com/PR9/A212).

### 3.7. Case studies of CYD2D6 poor metabolism

Four individuals who used oxycodone were found to be poor metabolizers, all self-identified as Anglo-American.^10,13^ One individual had a maximum pain of 4, which reduced to a 0 with oxycodone use; no adverse events were experienced. The next individual had maximum pain of 4, which reduced to a 2; constipation, drowsiness, nausea, and vomiting were reported. The next had maximum pain of 8, which reduced to a 4; drowsiness, nausea, and vomiting were reported. The last selected individual had maximum pain of 6, which reduced to a 2; they reported appetite loss, constipation, drowsiness, dizziness, nausea, and rash.

### 3.8. Case studies of possible CYD2D6 ultrarapid metabolism

Three individuals who used oxycodone were found to have potential ultrarapid metabolism, based on the presence of copy number variations for *1 and *41: 2 self-identified as Anglo-American and 1 as Indigenous. One individual had a maximum pain of 8, which reduced to a 0; adverse events included dizziness and drowsiness. The next individual had a maximum pain of 6, which reduced to 2; appetite loss and nausea were reported. The last had a maximum pain of 10, which reduced to a 6; no adverse events were experienced.

## 4. Discussion

Just over one quarter of children had an allelic variation known to be implicated in clinical outcomes for CYP2C9, whereas over one-third had “nonnormal” phenotype classification for CYP2D6. Decreased CYP2C9 function alleles (*2 and *3) were found in over one quarter of individuals. No impact on clinical analgesic effectiveness was found with ibuprofen use, but children who were carriers of *2 experienced less adverse events with ibuprofen; this contrasted our original hypothesis that decreased clearance would lead to higher concentrations of ibuprofen, predisposing to adverse events. Decreased CYP3A4 functioning alleles were found in 3.4% of individuals, whereas 36.5% had a decreased functioning phenotype for CYP2D6. We did not find an effect on analgesic effect or occurrence of adverse events with oxycodone use.

Different alleles implicated in drug metabolism can predispose individuals to faster or slower metabolism of various analgesics.^5,26^ Variants may lead to decreased medication clearance and increase risk of drug toxicity or other adverse events,^44^ such as gastric bleeding after NSAID use^25^; conversely, for prodrugs requiring activation, poor metabolism may lead to insufficient pain relief.^4^ The CYP2D6 normal metabolizer phenotype is known to be most common.^6^ Poor metabolizer status is more common among those of Anglo-American descent, with about 10% prevalence^11^; our study demonstrated 7.5% prevalence. Conversely, ultrarapid metabolism is uncommon in those of Anglo-American descent but more prevalent in East Africa, found in up to 29% of those with East African descent.^26,41^ Our study, representing a mostly Anglo-American population, supports these prior characterizations, with our participants found to be mostly CYP2D6 normal metabolizers but also having some representation of poor and intermediate metabolism. Although some prior studies have shown that oxycodone metabolite contribution to overall analgesic effect is affected by CYP2D6 phenotype,^38^ other studies have suggested that most analgesic effect comes from the parent compound oxycodone, rather than metabolites that are found in much lower concentrations.^27,29^ Our findings of no impact of CYP2D6 phenotype classification on clinical effect is congruent with the latter. Thus, our small study would imply that a child's CYP2D6 phenotype classification may not influence their response to oxycodone. This must be confirmed with larger scale studies and randomized allocation of participants.

CYP2C9 is less extensively characterized than CYP2D6, with allelic variants' function categorized as no function, decreased function, normal function, or unknown function based on currently described variants catalogued in genomic databases.^8,16^ CYP2C9*2 and *3 alleles are associated with poor metabolism and reduced clearance of NSAIDs and are known to be more common in those of Anglo-American descent,^12,44^ affecting up to 20%^21^; this slow clearance may put patients at higher risk for adverse events from ibuprofen.^31^ Our study reaffirmed this distribution, with slightly over 25% of our mostly Anglo-American population having those decreased functioning alleles. Prior study of CYP2C9 in adults has demonstrated that those with decreased functioning alleles are at an increased risk for developing acute gastrointestinal bleeding after taking ibuprofen and other NSAIDs.^1,32^ Furthermore, both the *2 and *3 have been previously demonstrated in adults to be associated with up to a 10-fold decrease in ibuprofen clearance (and thus increased clinical pain relief).^18^ By contrast, our study demonstrated no impact of CYP2C9 allelic variations on clinical analgesic effectiveness, and children who were carriers of *2 experienced *less* adverse events with ibuprofen, instead of the expected increase, based on adult studies. These findings merit further exploration with a larger cohort of participants as it is possible that these results are due to our smaller sample size or, perhaps, that children's metabolism and clinical effects of ibuprofen are influenced by more than pharmacogenetics, alone.

CYP3A4 serves as the primary metabolizer of oxycodone, converting it to noroxycodone.^23^ Allelic variation has been noted in particular genetic ancestry groups, with variants such as CYP3A4*1B—which may have increased enzyme activity^35^—found in 2% to 10% of Anglo-Americans, 9% to 11% of Hispanics, and 35% to 67% of African-Americans populations^35^; increased enzyme activity may lead to less analgesia as more oxycodone is converted to its inactive metabolite, noroxycodone.^23^ Our population had no participants with increased functioning alleles; rather, 4 participants had a reduced functioning allele. No clinical correlations with clinical effectiveness or adverse events were noted in our small study cohort who received oxycodone, precluding definitive conclusions in this regard.

A recent large translational study of adults has shown that providing adults with a QR code that stored the participants' test results for a 12 gene–drug interaction panel resulted in a 7% lower incidence of adverse events whenever a new medication was prescribed.^42^ Although personalized medicine based in pharmacogenomic profiles has clear clinical considerations for some cardiac medications (clopidogrel and warfarin)^7,39^ and chemotherapy (tamoxifen),^19^ its larger role in pain care, particularly for children, remains unclear at this time. Codeine is one notable exception, where an understanding of its metabolism has led to national recommendations to avoid its use because of the potential for fatal overdose in ultrarapid metabolizers.^20,45^ A recent study has shown that even when genomic results are made available to adults, they still do not reliably alter the use of medications such as ibuprofen, presumably reflecting other priorities beside avoidance of adverse events.^46^ It is important to note that genetic factors do not account for all of the variability in drug response and that genetic contribution varies not only between individual drugs but also between individual patients.^36^ As such, although pharmacogenomics of ibuprofen and other analgesics should continue to be studied and evaluated, the knowledge base is not necessarily at a point where it can be uniformly adopted into clinical practice.

The clinical implications of this study, when compared with other larger studies and recent reviews, are consistent with our current understanding of analgesic literature. Our study suggests that children prescribed ibuprofen or oxycodone experienced comparable pain relief, with the oxycodone cohort experiencing significantly more cumulative adverse effects over the study period of 3 days. In our team's primary analysis of this study cohort, which included over 300 children, we demonstrated similar pain relief between study drug cohorts, with the ibuprofen cohort experiencing 20% less adverse effects than the oxycodone cohort on day 1.^3^ Two systematic reviews of analgesia for children with musculoskeletal injuries have also demonstrated that ibuprofen is the preferable medication (over acetaminophen and oral opioids), given it similar analgesic properties to midpotency oral opioids such as oral morphine and oxycodone, with a far more desirable adverse effect profile.^34^ Current adult literature on ibuprofen use and genotype suggests a dosage reduction for ibuprofen in poor metabolizers; however, evidence is lacking to make this recommendation for children at this time.^43^ In the absence of compelling and available pharmacogenetic information for individual analgesic prescribing for children, the safest clinical choice would be to use ibuprofen in manufacturer-recommended doses (10 mg/kg//dose, maximum 600 mg/dose), for the best effectiveness to risk balance.

There are some limitations to our study*.* Inclusion criteria for the genomic association analyses, namely, requiring medication to be used on day 1, may have skewed selection towards patients with more painful fractures; however, all participants had nonoperative fractures and were discharged home from the ED. As the dosage and schedule of pain medication administration at home was based on family discretion, if allelic variants have clinical impact only at certain thresholds of drug concentration, this would not have been captured. Some families, particularly those using oxycodone, also used concomitant acetaminophen. Although this may seem a confounder for presented results, this study is a pragmatic observational cohort, and the use of acetaminophen with oxycodone reflects real-world use and recommendations for nonopioid cotherapy with oral opioid administration. As not every family used their prescribed medication on day 1, clinical results regarding efficacy and adverse events alone (Tables 3–5) should be interpreted with caution; notably, prior study of over 300 children using these same medications have yielded similar results.^2^ The FPS-R was used for participants aged 4 to 16 years; although it is formally recommended for 4 to 12 years, we extended this upper limit to allow for between-participant comparisons and greater generalizability. The small sample size for the oxycodone cohort precluded definitive conclusions from being drawn for the 3A4 variant. Small sample size for many genomic variants in our population precluded meaningful analysis of less-common polymorphisms. Furthermore, genomic assays cannot test for all possible CYP variants, necessitating assumption of “wild-type” status if no tested variants were found. Many polymorphisms remain of unknown significance at this time, and the expression of CYP is complex and likely affected by other variants outside the genes studied. This study's majority population self-identified as Anglo-American, so results may not be generalizable to other populations.

## 5. Conclusion

To personalize and best treat children's pain, one must understand the *clinically relevant* impact of variation in metabolic phenotypes on pain treatment. One quarter of study participants had an allelic variation known to be implicated in clinical outcomes for CYP2C9 and 7.2% for CYP3A4, whereas 36.5% had “nonnormal” phenotype classification for CYP2D6. Our results suggest that CYP2D6 phenotype classification and the CYP3A4 allelic variants that we studied did not affect the clinical effectiveness or adverse events associated with oxycodone use. Our study also suggests that children with a CYP2C9*2 variant allele who received ibuprofen had fewer adverse event occurrence, which is in contradiction to previously published adult studies. Given our relatively small sample size, these results must be confirmed with a larger sample to further characterize and confirm relationships between allelic variations and clinical outcomes in children, and to understand the impact of rarer CYP variants. Further understanding of pharmacogenetics may allow for individually tailored drug choice, minimizing adverse drug reactions and optimizing reliable medication response. Ultimately, progressing beyond a “one size fits all” method of selecting analgesic agents could further support personalized medicine.

## Disclosures

The authors have no conflict of interest to declare.

## Appendix A. Supplemental digital content

Supplemental digital content associated with this article can be found online at http://links.lww.com/PR9/A212.

## Supplementary Material

## References

[1] Agúndez JA García-Martín E Martínez C. Genetically based impairment in CYP2C8- and CYP2C9-dependent NSAID metabolism as a risk factor for gastrointestinal bleeding: is a combination of pharmacogenomics and metabolomics required to improve personalized medicine? Expert Opin Drug Metab Toxicol 2009;5:607–20. 19422321 10.1517/17425250902970998

[2] Ali S Drendel AL Kircher J Beno S. Pain management of musculoskeletal injuries in children: current state and future directions. Pediatr Emerg Care 2010;26:518–24; quiz 525–8. 20622635 10.1097/PEC.0b013e3181e5c02b

[3] Ali S Manaloor R Johnson D Rosychuk RJ LeMay S Carleton B McGrath PJ Drendel AL; Pediatric Emergency Research Canada. An observational cohort study comparing ibuprofen and oxycodone in children with fractures. PLoS One 2021;16:e0257021. 34499688 10.1371/journal.pone.0257021 PMC8428788

[4] Bailey B Trottier ED. Managing pediatric pain in the emergency department. Paediatr Drugs 2016;18:287–301. 27260499 10.1007/s40272-016-0181-5

[5] Balyan R Mecoli M Venkatasubramanian R Chidambaran V Kamos N Clay S Moore DL Mavi J Glover CD Szmuk P Vinks A Sadhasivam S. CYP2D6 pharmacogenetic and oxycodone pharmacokinetic association study in pediatric surgical patients. Pharmacogenomics 2017;18:337–48. 28244808 10.2217/pgs-2016-0183 PMC5558529

[6] Bernard S Neville KA Nguyen AT Flockhart DA. Interethnic differences in genetic polymorphisms of CYP2D6 in the U.S. population: clinical implications. Oncologist 2006;11:126–35. 16476833 10.1634/theoncologist.11-2-126

[7] Bourgeois S Jorgensen A Zhang EJ Hanson A Gillman MS Bumpstead S Toh CH Williamson P Daly AK Kamali F Deloukas P Pirmohamed M. A multi-factorial analysis of response to warfarin in a UK prospective cohort. Genome Med 2016;8:2. 26739746 10.1186/s13073-015-0255-y PMC4702374

[8] Cariaso M Lennon G. SNPedia: a wiki supporting personal genome annotation, interpretation and analysis. Nucleic Acids Res 2012;40:D1308–12. 22140107 10.1093/nar/gkr798 PMC3245045

[9] Carleton BC Poole RL Smith MA Leeder JS Ghannadan R Ross CJD Phillips MS Hayden MR. Adverse drug reaction active surveillance: developing a national network in Canada's children's hospitals. Pharmacoepidemiol Drug Saf 2009;18:713–21. 19507171 10.1002/pds.1772

[10] Caudle KE Sangkuhl K Whirl-Carrillo M Swen JJ Haidar CE Klein TE Gammal RS Relling MV Scott SA Hertz DL Guchelaar H-J Gaedigk A. Standardizing CYP2D6 genotype to phenotype translation: consensus recommendations from the clinical pharmacogenetics implementation consortium and Dutch pharmacogenetics working group. Clin Transl Sci 2020;13:116–24. 31647186 10.1111/cts.12692 PMC6951851

[11] Chidambaran V Sadhasivam S Mahmoud M. Codeine and opioid metabolism: implications and alternatives for pediatric pain management. Curr Opin Anaesthesiol 2017;30:349–56. 28323671 10.1097/ACO.0000000000000455 PMC5482206

[12] Cregg R Russo G Gubbay A Branford R Sato H. Pharmacogenetics of analgesic drugs. Br J Pain 2013;7:189–208. 26516523 10.1177/2049463713507439 PMC4590162

[13] Crews KR Gaedigk A Dunnenberger HM Leeder JS Klein TE Caudle KE Haidar CE Shen DD Callaghan JT Sadhasivam S Prows CA Kharasch ED Skaar TC; Clinical Pharmacogenetics Implementation Consortium. Clinical pharmacogenetics implementation consortium guidelines for cytochrome P450 2D6 genotype and codeine therapy: 2014 update. Clin Pharmacol Ther 2014;95:376–82. 24458010 10.1038/clpt.2013.254 PMC3975212

[14] Drendel A. Pharmacogenomics of analgesic agents. Clin Pediatr Emerg Med 2007;8:262–7.

[15] Elens L Norman E Matic M Rane A Fellman V Van Schaik RHN. Genetic predisposition to poor opioid response in preterm infants: impact of KCNJ6 and COMT polymorphisms on pain relief after endotracheal intubation. Ther Drug Monit 2016;38:525–33. 27027462 10.1097/FTD.0000000000000301

[16] Gaedigk A Ingelman-Sundberg M Miller N Leeder S Whirl-Carrillo M Klein TE; PharmVar Steering Committee. The pharmacogene variation (PharmVar) consortium: incorporation of the human cytochrome P450 (CYP) allele nomenclature database. Clin Pharmacol Ther 2018;103:399–401. 29134625 10.1002/cpt.910 PMC5836850

[17] Gaglani A Gross T. Pediatric pain management. Emerg Med Clin North Am 2018;36:323–34. 29622325 10.1016/j.emc.2017.12.002

[18] Garciamartin E Martínez C Tabarés B Frías J Agúndez JAG. Interindividual variability in ibuprofen pharmacokinetics is related to interaction of cytochrome P450 2C8 and 2C9 amino acid polymorphisms. Clin Pharmacol Ther 2004;76:119–27. 15289789 10.1016/j.clpt.2004.04.006

[19] Goetz MP Sangkuhl K Guchelaar HJ Schwab M Province M Whirl-Carrillo M Symmans WF McLeod HL Ratain MJ Zembutsu H Gaedigk A van Schaik RH Ingle JN Caudle KE Klein TE. Clinical pharmacogenetics implementation consortium (CPIC) guideline for CYP2D6 and tamoxifen therapy. Clin Pharmacol Ther 2018;103:770–7. 29385237 10.1002/cpt.1007 PMC5931215

[20] Government of Canada. Available at: https://healthycanadians.gc.ca/recall-alert-rappel-avis/hc-sc/2016/59584a-eng.php?_. Accessed May 25, 2023.

[21] Hersh EV Pinto A Moore PA. Adverse drug interactions involving common prescription and over-the-counter analgesic agents. Clin Ther 2007;29(suppl l):2477–97. 18164916 10.1016/j.clinthera.2007.12.003

[22] Hicks CL Von Baeyer CL Spafford PA Van Korlaar I Goodenough B. The Faces Pain Scale—revised: toward a common metric in pediatric pain measurement. PAIN 2001;93:173–83. 11427329 10.1016/S0304-3959(01)00314-1

[23] Huddart R Clarke M Altman RB Klein TE. PharmGKB summary: oxycodone pathway, pharmacokinetics. Pharmacogenet Genomics 2018;28:230–7. 30222708 10.1097/FPC.0000000000000351 PMC6602093

[24] International Council for Harmonisation of Technical Requirements for Pharmaceuticals for Human Use (ICH). Medical dictionary for regulatory activities (MedDRA). Available at: https://www.meddra.org/. Accessed July 22, 2020.

[25] Jaja C Bowman L Wells L Patel N Xu H Lyon M Kutlar A. Preemptive genotyping of CYP2C8 and CYP2C9 allelic variants involved in NSAIDs metabolism for sickle cell disease pain management. Clin Transl Sci 2015;8:272–80. 25640739 10.1111/cts.12260 PMC4522406

[26] Jimenez N Galinkin JL. Personalizing pediatric pain medicine: using population-specific pharmacogenetics, genomics, and other-omics approaches to predict response. Anesth Analg 2015;121:183–7. 26086515 10.1213/ANE.0000000000000721

[27] Klimas R Witticke D El Fallah S Mikus G. Contribution of oxycodone and its metabolites to the overall analgesic effect after oxycodone administration. Expert Opin Drug Metab Toxicol 2013;9:517–28. 23488585 10.1517/17425255.2013.779669

[28] Koren G Cairns J Chitayat D Gaedigk A Leeder SJ. Pharmacogenetics of morphine poisoning in a breastfed neonate of a codeine-prescribed mother. Lancet 2006;368:704. 16920476 10.1016/S0140-6736(06)69255-6

[29] Lalovic B Kharasch E Hoffer C Risler L Liu-Chen LY Shen DD. Pharmacokinetics and pharmacodynamics of oral oxycodone in healthy human subjects: role of circulating active metabolites. Clin Pharmacol Ther 2006;79:461–79. 16678548 10.1016/j.clpt.2006.01.009

[30] Le May S Ali S Plint AC Masse B Neto G Auclair M-C Drendel AL Ballard A Khadra C Villeneuve E Parent S McGrath PJ Leclair G Gouin S; Pediatric Emergency Research Canada PERC. Oral analgesics utilization for children with musculoskeletal injury (OUCH Trial): an RCT. Pediatrics 2017;140:e20170186. 29021235 10.1542/peds.2017-0186

[31] Mazaleuskaya LL Theken KN Gong L Thorn CF FitzGerald GA Altman RB Klein TE. PharmGKB summary: ibuprofen pathways. Pharmacogenet Genomics 2015;25:96–106. 25502615 10.1097/FPC.0000000000000113 PMC4355401

[32] Michels SL Collins J Reynolds MW Abramsky S Paredes-Diaz A McCarberg B. Over-the-counter ibuprofen and risk of gastrointestinal bleeding complications: a systematic literature review. Curr Med Res Opin 2012;28:89–99. 22017233 10.1185/03007995.2011.633990

[33] OpenClinica LLC. Available at: https://www.openclinica.com/. Accessed November 30, 2020.

[34] Parri N Lazzeri S. Efficacy of ibuprofen in musculoskeletal post-traumatic pain in children: a systematic review. PLoS One 2020;15:e0243314. 33270748 10.1371/journal.pone.0243314 PMC7714211

[35] Pergolizzi JV Taylor R LeQuang JA Raffa RB Breve F. Towards personalized opioid dosing: a concise overview of CYP polymorphisms. Anesthesiol Clin Sci Res 2018;2:11–19.

[36] Pirmohamed M. Pharmacogenomics: current status and future perspectives. Nat Rev Genet 2023;24:350–62. 36707729 10.1038/s41576-022-00572-8

[37] R: a language and environment for statistical computing. R R Proj Stat Comput. 2019. Available at: https://www.r-project.org/. Accessed August 3, 2020.

[38] Samer CF Daali Y Wagner M Hopfgartner G Eap CB Rebsamen MC Rossier MF Hochstrasser D Dayer P Desmeules JA. The effects of CYP2D6 and CYP3A activities on the pharmacokinetics of immediate release oxycodone. Br J Pharmacol 2010;160:907–18. 20590587 10.1111/j.1476-5381.2010.00673.x PMC2935997

[39] Shuldiner AR O'Connell JR Bliden KP Gandhi A Ryan K Horenstein RB Damcott CM Pakyz R Tantry US Gibson Q Pollin TI Post W Parsa A Mitchell BD Faraday N Herzog W Gurbel PA. Association of cytochrome P450 2C19 genotype with the antiplatelet effect and clinical efficacy of clopidogrel therapy. JAMA 2009;302:849–57. 19706858 10.1001/jama.2009.1232 PMC3641569

[40] Sistonen J Fuselli S Levo A Sajantila A. CYP2D6 genotyping by a multiplex primer extension reaction. Clin Chem 2005;51:1291–5. 15905314 10.1373/clinchem.2004.046466

[41] Sistonen J Sajantila A Lao O Corander J Barbujani G Fuselli S. CYP2D6 worldwide genetic variation shows high frequency of altered activity variants and no continental structure. Pharmacogenet Genomics 2007;17:93–101. 17301689 10.1097/01.fpc.0000239974.69464.f2

[42] Swen JJ van der Wouden CH Manson LE Abdullah-Koolmees H Blagec K Blagus T Böhringer S Cambon-Thomsen A Cecchin E Cheung KC Deneer VH Dupui M Ingelman-Sundberg M Jonsson S Joefield-Roka C Just KS Karlsson MO Konta L Koopmann R Kriek M Lehr T Mitropoulou C Rial-Sebbag E Rollinson V Roncato R Samwald M Schaeffeler E Skokou M Schwab M Steinberger D Stingl JC Tremmel R Turner RM van Rhenen MH Dávila Fajardo CL Dolžan V Patrinos GP Pirmohamed M Sunder-Plassmann G Toffoli G Guchelaar HJ; Ubiquitous Pharmacogenomics Consortium. A 12-gene pharmacogenetic panel to prevent adverse drug reactions: an open-label, multicentre, controlled, cluster-randomised crossover implementation study. Lancet 2023;401:347–56. 36739136 10.1016/S0140-6736(22)01841-4

[43] Theken KN Lee CR Gong L Caudle KE Formea CM Gaedigk A Klein TE Agúndez JAG Grosser T. Clinical pharmacogenetics implementation consortium guideline (CPIC) for CYP2C9 and nonsteroidal anti-inflammatory drugs. Clin Pharmacol Ther 2020;108:191–200. 32189324 10.1002/cpt.1830 PMC8080882

[44] Ting S Schug S. The pharmacogenomics of pain management: prospects for personalized medicine. J Pain Res 2016;9:49–56. 26929662 10.2147/JPR.S55595 PMC4755469

[45] U.S. Food and Drug Administration. Available at: https://www.fda.gov/drugs/drug-safety-and-availability/fda-drug-safety-communication-fda-restricts-use-prescription-codeine-pain-and-cough-medicines-and. Accessed May 25, 2023.

[46] Zajic SC Jarvis JP Zhang P Rajula KD Brangan A Brenner R Dempsey MP Christman MF. Individuals with CYP2C8 and CYP2C9 reduced metabolism haplotypes self-adjusted ibuprofen dose in the Coriell Personalized Medicine Collaborative. Pharmacogenet Genomics 2019;29:49–57. 30562214 10.1097/FPC.0000000000000364

[47] Zhang H Chen M Wang X Yu S. Patients with CYP3A4∗1G genetic polymorphism consumed significantly lower amount of sufentanil in general anesthesia during lung resection. Medicine 2017;96:e6013. 28121959 10.1097/MD.0000000000006013 PMC5287983

