# Supplementary material for: Effects of pharmacogenetic profiles on pediatric pain relief and adverse events with ibuprofen and oxycodone
Source: Pain Rep. 2023 Oct 17;8(6):e1113. doi: 10.1097/PR9.0000000000001113 (PMC10659733; doi:10.1097/PR9.0000000000001113)
Supplement: Supplementary file 1 [file painreports-8-e1113-s001.pdf]

Supplemental Table 1: At-Home Pharmacologic Pain Management Used

	Ibuprofen Group (n=140) n (%)			Oxycodone Group (n=70) n (%)			Total Cohort (n=210) n (%)		
	Day 1	Day 2	Day 3	Day 1	Day 2	Day 3	Day 1	Day 2	Day 3
**Ibuprofen Only**	139 (99.3)	108 (77.1)	76 (54.3)	10 (14.3)	10 (14.3)	11 (15.7)	149 (71.0)	118 (56.2)	87 (41.4)
**Oxycodone Only**	1 (0.7)	0 (0.0)	1 (0.7)	13 (18.6)	10 (14.3)	10 (14.3)	14 (6.7)	10 (4.8)	11 (5.2)
**Acetaminophen Only**	0 (0)	3 (2.1)	1 (0.7)	0 (0)	10 (14.3)	12 (17.1)	0 (0.0)	13 (6.2)	13 (6.2)
**Ibuprofen and Acetaminophen**	0 (0)	6 (4.3)	4 (2.9)	0 (0)	0 (0)	0 (0)	1 (0.5)	6 (2.9)	4 (1.9)
**Acetaminophen + Oxycodone Combination Tablet***	0 (0)	0 (0)	0 (0)	47 (67.1)	32 (45.7)	17 (24.3)	47 (22.4)	32 (15.2)	17 (8.1)
**None** †	N/A	23 (16.4)	58 (41.4)	N/A	8 (11.4)	20 (28.6)	N/A	31 (14.8)	78 (37.1)

*Oxycodone Group: Day 1 (n=1 also used codeine); Day 2 (n=1 also used codeine; n=2 also used ibuprofen); Day 3 (n=2 also used ibuprofen).

† Inclusion criteria required patients to have taken medication on Day 1.

Supplemental Table 2. Adverse Events on Days 1-3 by Treatment Group

**Day 1**		**Ibuprofen Group (n=140) n (%)**	**Oxycodone Group (n=69) n (%)**	**Total Cohort (n=208) n (%)**	**p Value**
	**Any AE**	74 (52.9)	54 (78.3)	128 (61.6)	<0.001
	**Abdominal Pain**	7 (5.0)	6 (8.7)	13 (6.2)	0.30
	**Appetite Loss**	34 (24.3)	26 (37.7)	60 (28.8)	**0.04**
	**Constipation**	6 (4.3)	12 (17.4)	18 (8.7)	**0.002**
	**Dizziness**	11 (7.9)	18 (26.1)	29 (13.9)	**<0.001**
	**Drowsiness**	56 (40.0)	44 (63.8)	100 (48.1)	**0.001**
	**Nausea**	13 (9.3)	21 (30.4)	34 (16.3)	**<0.001**
	**Rash**	3 (2.1)	1 (1.4)	4 (1.9)	0.73
	**Vomiting**	3 (2.1)	7 (10.1)	10 (4.8)	0.01
**Day 2**		**Ibuprofen Group (n=140) n (%)**	**Oxycodone Group (n=66) n (%)**	**Total Cohort (n=206) n (%)**	**p Value**
	**Any AE**	50 (35.7)	38 (57.6)	88 (42.7)	0.003
	**Abdominal Pain**	3 (2.1)	7 (10.6)	10 (4.9)	**0.008**
	**Appetite Loss**	21 (15.0)	15 (22.7)	36 (17.5)	0.17
	**Constipation**	6 (4.3)	4 (6.1)	10 (4.9)	0.58
	**Dizziness**	4 (2.9)	10 (15.2)	14 (6.8)	**0.001**

	**Drowsiness**	30 (21.4)	30 (45.5)	60 (29.1)	**<0.001**
	**Nausea**	6 (4.3)	10 (15.2)	16 (7.8)	**0.007**
	**Rash**	0 (0.0)	3 (4.5)	3 (1.5)	**0.01**
	**Vomiting**	0 (0.0)	1 (1.5)	1 (0.5)	0.14
**Day 3**		**Ibuprofen Group (n=140) n (%)**	**Oxycodone Group (n=64) n (%)**	**Total Cohort (n=204) n (%)**	**p Value**
	**All**	31 (22.1)	21 (32.8)	55 (27.0)	0.10
	**Abdominal Pain**	4 (2.9)	4 (6.2)	8 (3.9)	0.25
	**Appetite Loss**	13 (9.3)	9 (14.1)	22 (10.8)	0.31
	**Constipation**	7 (5.0)	3 (4.7)	10 (4.9)	0.92
	**Dizziness**	5 (3.6)	7 (10.9)	12 (5.9)	**0.04**
	**Drowsiness**	17 (12.1)	13 (20.3)	30 (14.7)	0.13
	**Nausea**	5 (3.6)	6 (9.4)	11 (5.4)	0.09
	**Rash**	1 (0.7)	2 (3.1)	3 (1.5)	0.18
	**Vomiting**	0 (0.0)	3 (4.7)	3 (1.5)	**0.01**

Supplemental Table 3a. Estimates of Genomic Associations with Pain Reduction

Variable	Estimate	Standard Error	p value
**CYP2C9**			
CYP2C9*2	0.60	0.48	0.22
CYP2C9*3	-0.79	0.59	0.18
CYP2C9*6	-1.52	1.84	0.41
**CYP2D6**			
Poor Metabolizer	-0.36	1.23	0.77
Intermediate Metabolizer	-1.42	0.73	0.06
**CYP3A4**			
CYP3A4_20239G>A	-0.15	0.69	0.83
CYP3A4*1B	-0.11	0.86	0.90

Supplemental Table 3b. Estimates of Genomic Associations with Adverse Events

Variable	OR (95% CI)	p value
**CYP2C9**		
**CYP2C9*2**	**0.72 (0.58, 0.89)**	**0.003**
CYP2C9*3	0.80 (0.60, 1.05)	0.11
CYP2C9*6	0.54 (0.21, 1.41)	0.21
**CYP2D6**		
Poor Metabolizer	0.96 (0.65, 1.43)	0.85
Intermediate Metabolizer	1.16 (0.93, 1.46)	0.20
**CYP3A4**		
CYP3A4*_20239G>A	0.98 (0.77, 1.25)	0.88
CYP3A4*1B	0.97 (0.73, 1.29)	0.84

Supplemental Table 4a. Estimates of Genomic Associations with Pain Reduction Adjusted by Clinical Factors

Variable	Estimate	Standard Error	p value
**Ibuprofen**			
Age	-0.05	0.05	0.30
Sex	0.12	0.37	0.76
Fracture Location	-0.44	0.43	0.31
Fracture Reduction	-0.08	0.38	0.83
Non-Pharmacologic Management	-1.34	1.10	0.23
Ethnicity	0.05	0.26	0.86
CYP2C9*2	0.66	0.50	0.19
CYP2C9*3	-0.79	0.62	0.21
CYP2C9*6	-1.55	1.93	0.42
**Oxycodone – CYP2D6**			
Age	-0.26	0.14	0.06
Sex	-1.81	0.90	0.05
Fracture Location	-1.05	0.97	0.29
Fracture Reduction	-0.73	0.74	0.31
**Non-Pharmacologic Management**	**-4.56**	**1.82**	**0.02**
Ethnicity	0.04	0.92	0.96
CYP2D6 Intermediate	0.84	1.34	0.53
CYP2D6 Normal	2.18	1.28	0.10

Supplemental Table 4b. Estimates of Genomic Associations with Adverse Events Adjusted by Clinical Factors

Variable	OR (95% CI)	p value
**Ibuprofen**		
Age	1.00 (0.97, 1.02)	0.76
Sex	0.92 (0.78, 1.09)	0.35
Fracture Location	1.14 (0.93, 1.39)	0.21
Sedation in ED	1.07 (0.89, 1.28)	0.50
Ethnicity	0.98 (0.88, 1.10)	0.77
**CYP2C9*2**	**0.73 (0.58, 0.90)**	**0.005**
CYP2C9*3	0.78 (0.58, 1.04)	0.09
CYP2C9*6	0.57 (0.21, 1.54)	0.27
**Oxycodone – CYP2D6**		
Age	0.97 (0.93, 1.02)	0.24
Sex	1.24 (0.93, 1.66)	0.15
Fracture Location	1.12 (0.82, 1.52)	0.48
Sedation in ED	1.05 (0.83, 1.33)	0.67
Ethnicity	1.36 (0.99, 1.87)	0.07
CYP2D6 Intermediate	1.09 (0.69, 1.71)	0.71
CYP2D6 Normal	0.90 (0.58, 1.39)	0.64
